# New products

**DOI:** 10.1155/S1463924685000359

**Published:** 1985


Journal of Automatic Chemistry/*Journal of Clinical Laboratory Automation*, Vol. 7, No. 3 (Jul–Sep 1985), pp. 160–168

### New products

### PITTSBURGH PRODUCT REVIEW

### About the 36th International Pittsburgh Conference & Exposition on Analytical Chemistry and Applied Spectroscopy

The 1985 Pittsburgh meeting was held at the New Orleans Convention Center in New Orleans, Louisiana, USA from 25 February to 1 March. 660 companies exhibited (in 1850 booths) which represented a 15·1% increase on commercial participation over the 1984 conference and exposition. Some of the products launched at Pittsburgh are described in this review.

Symposia held at the 36th Pittsburgh Conference were:

ASTM E-42 small area solid and surface analysis – arranged by R. J. Colton, Naval Research Laboratory and S. W. Gaarenstroom, General Motors Research Laboratories.

Toxic waste analysis/water pollution analysis – arranged by H. V. Drushel, Exxon Research and Development Laboratories.

Near infra-red analysis – arranged by J. G. Montalvo, US Department of Agriculture.

New dimensions in particle size and shape technologies – arranged by J. W. Novak, Jr., Alcoa Laboratories.

Flow-injection analysis – arranged by J. F. Coetzee, University of Pittsburgh and H. A. Mottola, Oklahoma State University.

New advances in Raman spectroscopy – arranged by S. A. Asher, University of Pittsburgh.

Analytical applications of micellar solutions – arranged by J. G. Dorsey, University of Florida and L. J. Cline Love, Seton Hall University.

Reaction detectors in liquid chromatography – arranged by R. Weinberger, Kratos Analytical Instruments.

Application of optical fibres and lasers to chemical analysis – arranged by G. M. Hieftje, Indiana University.

Computers in analytical instrumentation: towards intelligent tools for the laboratory – arranged by J. P. Avery, University of Colorado and S. R. Crouch, Michigan State University.

Planar chromatography – arranged by R. E. Kaiser, Chromatographic Institute and H. M. Stahr, Iowa State University.

Biomedical infra-red spectroscopy: the biomedical approach – arranged by R. J. Jakobsen, Battelle-Columbus Laboratories.

Advances in mass spectrometry – arranged by A. G. Sharkey, Pittsburgh Energy Technology Center/US DOE.

Microbore liquid chromatography – arranged by J. Q. Walker, IBM Instruments.

The 1985 Pittsburgh Conference and Exposition is a well-known forum for the presentation of major awards to distinguished scientists:

(1) The Pittsburgh Spectroscopy Award, sponsored by the Spectroscopy Society of Pittsburgh was presented to Dr Peter R. Griffiths of the University of California.

(2) The Pittsburgh Analytical Chemistry Award, sponsored by the Society for Analytical Chemists of Pittsburgh, was presented to Dr Bruce R. Kowalski of the Laboratory for Chemometrics at the University of Washington.

(3) The Charles N. Reilley Award, given by the Society for Electroanalytical Chemistry, sponsored by Bioanalytical Systems, Inc. was presented to Dr Ralph N. Adams, University Distinguished Professor at the University of Kansas.

(4) The Williams-Wright Industrial Spectroscopy Award, sponsored by the Coblentz Society, to Dr Clara D. Craver of Chemir Laboratories and Richard A. Nyquist of the Dow Chemical Company.

(5) The Maurice F. Hasler Award, sponsored by Applied Research Laboratory Division of Bausch and Lomb, to Dr Paul C. Lauterbur of the State University of New York at Stony Brook.

*Continuing Education:*

The Pittsburgh Conference and Exposition again sponsored a series of courses as part of its technical programme for continuing education. The goal was to provide a variety of topics of special interest to analytical chemists and spectroscopists. The short course and mini-courses were:

*Ion chromatography* – presented by Hamish Small, 1975 recipient of the first Pittsburgh Analytical Chemistry Award and the 1984 winner of the Dal Nogare Award at the 1984 Pittsburgh Conference and Exposition.

*Statistics* – presented by Dr John Taylor of the National Bureau of Standards.

*Thermospray LC-MS* – presented by Dr Marv Vestal.

*Petrochemical analysis* – presented by Dr Harry Drushel of Exxon Corporation.

*Quality assurance* – presented by Dr John Taylor of the National Bureau of Standards.

*Column preparation* – presented by Dr Charles Lochmüller.

*Introduction to Chemometrics* – presented by Dr Bruce Kowalski of the University of Washington.

Workshop: *Personal computers in the laboratory* – presented by C. Enke, Michigan State University and J. Avery, University of Colorado.

*For information about 1986's Pittsburgh Conference contact Mrs Linda Briggs, Executive Secretary: The Pittsburgh Conference & Exposition, 437 Donald Road, Pittsburgh, Pennsylvania 15235, USA. Tel.: 412 795 7667.*

### Nelson Analytical

*(Lynne Weaver, 10061 Bubb Road, Cupertino, California 95014; tel.: 408 725 1107)*

*Release-rate software*

Nelson Analytical, Inc. has recently introduced a software package to reduce data from up to 216 release-rate experiments to a single table of results. Called the Model 2635 Release Rate Tables Software, this package has been designed to record data from the company's IBM PC-based chromatography data system. Typical applications for this software package include drug-monitoring applications:

Analysis of release rate.

Dissolution rate.

Metabolism rate.

Quality-assurance applications:

Comparing the concentration of a compound or several compounds in different production lots.
Monitoring the concentration of a compound in a chemical reaction.
Comparing the reaction yields using different synthetic techniques or reactants.
Monitoring the shelf life of a product.

The Release Rate Tables software reads processed area table files from Nelson Analytical's Model 2600 Chromatography Software and places the compound concentration data in a format convenient for comparising chromatographic results from many runs.

The tables of release rate data can be printed or stored in a disk file. The disk files can be entered into spreadsheet programs like LOTUS 1-2-3, to produce graphs or perform other calculations on the data.

*Simulated distilllation method software*

Simulated Distillation (SIMDIS) is a combined gas chromatography and software method used in the analysis of petroleum-based products. The recently introduced Nelson Analytical Model 2880 Simulated Distillation Software for the IBM PC provides data processing for complex SIMDIS chromatograms using the standard ASTM D2887 and D3710 methods. Chromatographic data is acquired using Nelson's A/D interface and standard chromatography software. After all the requested chromatographic reports have been printed by the PC, the SIMDIS program is automatically signalled to operate, and the necessary reports and plots are generated.

The primary report generated by this program is the Percent Off Table. The increment size between percent off values is user selected. The Percent Off Table can be printed, or stored on disk for further analysis or for transfer to another computer.

The SIMDIS program also can be run in the interactive Simulated Distillation Review mode. This allows the user to interactively select new base-lines or base-line types, and generate Percent Off tables and plots; the software is menu-driven.

*Software-driven network*

Up to four Nelson Analytical Series 4400X Chromatography Desktop Data Systems can be networked with the Model 5350 Sharesman Network System. The system consists of software for the Series 4400X (a system based on the HP 9000 Series 200 technical computer), the Sharesman Controller, and the necessary cabling. These data systems are networked to a common mass-storage device. All software on the common mass storage is shared between the individual data systems. The Sharesman Controller acts as a 'traffic cop' to regulate disk access and control. It assigns control of shared disks to the Series 4400X systems on a network for common reading and writing of methods, data, and results files.

Specific software routines are embedded in each of the Sharesman programs to permit multiple Series 4400X systems in the network to access, store, and retrieve files from the same Winchester disk drive. This sharing approach not only allows simultaneous use of the Winchester disk drive, but permits increased keyboard time so that different operators can analyse and interpret chromatographic data at different workstations. The system is fully compatible with 5-, 15-megabyte, and greater Winchester disk drives, including the Hewlett-Packard CS80 binary call-based drives which feature high speed and reliability. System expansion is possible through the addition of multiple data systems, peripherals, and A/D interfaces.

**Circle No. 72 on Reader Enquiry Card**

### FiAtron

(*John Guehlstorf, 6651 N. Sidney Place, Milwaukee, Wisconsin 53209; tel.: 414 351 6650*)

*Solution-handling system*

The SHS-300 has been designed to increase precision, accuracy, detection limit, and productivity. This is accomplished by using advanced flow injection analysis (FIA) techno ogy. Developed by FiAtron Systems, Milwaukee, the SHS-300 has microprocessor-controlled solution handling, and a programmable, optically-encoded pumping system. FIA allows for total peak integration of precise sample pulses. Automated recalibration and access to four different standard solutions, for on-stream standard addition afford increased accuracy. Such new techniques can deal with spectral and matrix interference problems commonly encountered in atomic spectroscopy.

Detection limit is improved by automated on-stream dilution of highly concentrated solutions, as well as steady-state, undiluted injection of trace analyte solutions. When this dual injection procedure is used in conjunction with a series of standards, *two* different calibration curves are generated – one utilized for samples of low analyte concentration, and the other for highly concentrated samples.

High sampling rates, along with controlled dispersion, is readily implemented. A fully programmable sample changer is also controlled by the SHS-300. An RS-232 port allows easy interface to ICP/DCP computers or terminals. FiAtron offers a hand-held terminal for easy start-up, and control software for AA is being developed.

**Circle No. 73 on Reader Enquiry Card**

### GCA Corporation

(*Gerald Cuzelis, 3737 West Cortland Street, Chicago, Illinois 60647; tel.: 312 227 2660*)

*Laboratory robot*

The capabilities of a robot were demonstrated at Pittsburgh. The robot simulated such laboratory procedures as sample preparation, analysis and processing in standard laboratories, as well as in bio-hazard and radioactive areas. An IBM Personal Computer communicates with the robot and collects data at various stages during the lab procedures. The robotic demonstration featured a GCA/DK 200V preparing a sample and weighing it in a Mettler balance and dispenser. The robot then placed the sample into one of GCA's new high-temperature mechanical (HTM) convection ovens, and later removed the sample. Also on display was an artist's concept of a 'laboratory of the future', showing a laboratory with a built-in, overhead gantry robot and another robot positioned on a horizontal track.

*Ovens*

The GCA/Instruments and Equipment Group introduced two new families of Precision laboratory ovens and a new Precision Microwave/ Convection Oven at the 1985 Pittsburgh Conference. The Precision laboratory ovens are designed for a variety of applications, including drying, curing and moisture analysis.

A generation of microprocessor-controlled, high-temperature mechanical (HTM) convection ovens is available in four sizes, ranging from 55 l to 150 l chamber capacity. These economical HTM ovens are priced from $1900 to $2525.

A new line of gravity convection (Model STG) and mechanical convection (Model STM) general-purpose ovens is available in three sizes each, ranging from 40 l to 145 l chamber capacity. Model STG ovens are priced from $900 to $1700. Model STM ovens range from $1200 to $2100.

The Precision Microwave/ Convection Oven, the first of its kind available to laboratories, offers the flexibility and advantages of both drying methods. This oven can be used separately for microwave heating or mechanically-convected heating, or as a combination oven using both heating systems simultaneously.

**Circle No. 74 on Reader Enquiry Card**

### IBM Instruments, Inc.

*(Martin Hamer, Orchard Park, PO Box 332, Danbury, Connecticut 06810; tel.: 203 796 2654)*

IBM Instruments, Inc. had two significant new products at the Pittsburgh Conference to support its new 'integrated solutions' approach to analytical laboratory needs.

A two-oven gas chromatograph with a heart-cutting capability, the IBM GC 9630, has the ability to provide greatly improved sample resolution by transferring an unresolved sample fraction to a second column at a different temperature for a more complete separation.

For laboratories requiring a mid-range, bench-top Fourier Transform Infrared Spectrometer for repetitive analysis, the Company has introduced the IR/38 FTIR. It has a 4000-400 wavenumber energy range and true variable resolution to 1 wavenumber (apodized).

**Circle No. 75 on Reader Enquiry Card**

### Hewlett-Packard

*(Jean Hyams, 3000 Hanover Street, Palo Alto, California 94304; tel.: 415 857 5487)*

*HP and Nelson*

The first product resulting from HP's OEM agreement with Nelson Analytical, Inc. was announced at Pittsburgh. The product, a chromatography workstation, is designed to meet the data-acquisition, analysis and file-management needs of individual chemists or small laboratories. The standard configuration includes an HP 9000 Series 200 computer with substantial memory capacity, HP ThinkJet printer and 15-megabyte Winchester-disk drive from Hewlett-Packard, and XtraChrom chromatography software and an intelligent A/D interface made by Nelson Analytical.

*See also . 167*

**Circle No. 76 on Reader Enquiry Card**

### Nicolet Analytical Instruments

*(Jeffrey Christenson, 5225-1 Verona Road, PO Box 4508, Madison, Wisconsin 53711; tel.: 608 273 5004)*

*FT-IR*

The 5DXB Fourier transform infra-red (FT-IR) spectrometer was described as providing advanced capabilities at a modest price, along with available options and upgrades to accommodate future applications. Designed for the first-time FT-IR user making the transition from dispersive IR, the 5DXB allows complete spectral processing from a simple-to-use menu software system with detailed 'help' and advice information. The 5DXB incorporates a new optics bench designed for precise assembly and reliable performance. It includes a proprietary high-energy air-cooled source for enhanced sensitivity, and a rugged interferometer capable of 2 $cm^{-1}$ spectral resolution. An external beam can be computer-selected for special applications. The 5DXB data system is optimized for real-time spectral analysis and includes sophisticated multi-tasking software for fast spectral acquisition and processing. Options for enhanced performance include highest sensitivity MCT detectors, high-capacity Winchester SMD technology disk storage, and auxiliary experiment modules for special applications such as a high throughput microbeam for microsamples, a microscope for viewing and aligning samples, and an experiment module that can be optimized for dedicated experiments.

*Ultra-high resolution mass spectrometer*

Ultra-high resolution and exact mass measurements can be routinely achieved with the Nicolet FTMS-2000 Mass Spectrometer, over a wide mass range and under a variety of sampling and operating conditions including GC/MS, chemical ionization (for both positive and negative ions), as well as electron impact. This level of versatility is obtained without imposing the mechanical complexity of conventional mass spectrometers.

All functions of the new-generation FTMS instrument are entirely computer-controlled, using the Nicolet 1280 minicomputer and its new operating system, via sophisticated user-friendly software.

The standard system incorporates a superconducting magnet, a dual gas inlet system, a septum inlet system, an automatic solids probe inlet system, a dual high-speed pumping system, and a fully automated microprocessor-based vacuum system controller.

Available options include a capillary GC/MS interface for high-resolution mass analysis or large molecules such as biological compounds and polymers.

**Circle No. 77 on Reader Enquiry Card**

### Perkin-Elmer Corporation

(*Edward C. Collins, Main Avenue, Norwalk, Connecticut 06856; tel.: 203 6762 1000*)

Totally automated, high speed laboratory analysis with robotics, and the broadest line of analytical instruments and software in the industry were Perkin-Elmer's theme at the Pittsburgh Conference. The centrepiece of Perkin-Elmer's exhibit was a MasterLab with a robotic system capable of preparing samples for instrument analysis.

Nineteen major new instruments and accessories in seven analytical techniques, each designed to work in an automated laboratory environment, were demonstrated, as well as new analytical software for computer-aided chemistry.

The Perkin-Elmer *MasterLab System* employs a robot to transfer samples between laboratory work stations that weigh, transfer reagents, mix and perform other laboratory operations. Samples are then automatically transferred to an instrument for analysis.

The system is controlled by a personal computer and PERL (Perkin-Elmer Robot Language), which allows custom analytical preparation procedures to be easily programmed with simple commands. Communication with other laboratory instruments and computers is possible using an RS-232 interface

*Atomic spectroscopy*

The *Plasma II Inductively Coupled Plasma Emission Spectrometer* is an optimized sequential system for determining over 70 elements in a variety of sample matrices. It can perform automatic analysis at the optimum analytical wavelength for each element being determined in each sample being analysed. With an analytical throughput rate of up to 50 elements/min, the Plasma II provides the speed of a simultaneous system.

*Infra-red spectroscopy*

The *1700 Series Fourier Transform Infra-red Spectrometers* are based on a single high-performance interferometer. The two instruments in the 1700 Series, the Model 1710 and the Model 1750, differ primarily in their data handling capabilities.

New options and accessories are available for Perkin-Elmer's *Model 1800 FT-IR*, the industry's first double beam Fourier Transform Infra-red Spectrophotometer. They include: a *mercury cadmium telluride (MCT)* detector that will find applications in kinetics, forensic studies, trace analysis and difficult samples; a *second beamsplitter* that will extend the frequency range of the Model 1800 to allow the study of organometallic compounds, metal oxides, and catalyst research; a *photoacoustic detector accessory* that can be used for irregular shaped samples; an *infra-red microscope* that can obtain spectra of samples as small as 20 μm in diameter; and a *diffuse reflectance accessory* to obtain spectra from pharmaceuticals, foods, soaps, coal, clays and paints.

*Fluorescence spectroscopy*

The *Model 650-15 Fluorescence Spectrophotometer* is a low-cost, high-performance instrument designed for routine analytical work. It is especially useful as a simple, flexible and sensitive fluorescence spectrophotometer for quantitative purposes.

*Liquid chromatography*

The low-cost *3D HPLC System* is a completely functioning isocratic HPLC system with simultaneous UV, conductivity, and fluorescence detection. Employing Perkin-Elmer's TRI-DET (trifunctional detection), the system has application in ion chromatography and a variety of routine analyses.

The *Series 400 Liquid Chromatography System* is a cost effective quaternary solvent delivery system for liquid chromatography. Based on 16-bit microprocessor architecture, the Series 400 assures precise control of flow and solvent blending, offers excellent performance, and is exceptionally easy to use. Applications include the pharmaceutical, biomedical, environmental, and energy areas of HPLC analysis.

Perkin-Elmer's new *cartridge column system for liquid chromatography* offers chromatographers excellent column efficiencies, long column life, and ease of use at much lower cost than conventionally packed columns for liquid chromatography. The column packing has been reduced to its simplest and most economical form by use of replaceable cartridge that can be installed in less than 1 min.

*Gas chromatography*

Two multi-sampling systems are now available for Perkin-Elmer's Model 8300 Series gas chromatograph: the *Model AS-8300 Autosampler* for liquids (see p. 168), and the *Automatic thermal Desorption System* for adsorption tubes. Both are programmable multi-sampling systems that automate sample injection into any Model 8300 Gas Chromatograph.

A photometric detector (FPD) was announced for the SIGMA 2000 Gas Chromatograph. A major improvement in the *SIGMA 2000 FPD* is a minimum detectability of sulphur compounds of 5 to 10 times over previous models. The detector is sensitive to subnanogram quantities of sulfur and phosphorous compounds. The FPD is also highly selective for sulphur and phosphorous compounds, being 10 000 times less sensitive to hydrocarbons than sulphur compounds.

Perkin-Elmer's new *narrow bore (100 μm internal diameter) fused-silica bonded-phase capillary columns* are ideal for the analysis of petroleum and fuel oil samples. The columns provide high resolution gas chromatography with short analysis times.

*Thermal analysis*

The *TMA 7 Thermomechanical Analyzer* is the latest addition to the growing line of Perkin-Elmer 7 Series Thermal Analysis System instrument modules. Designed to complement other 7 Series instrument modules, which include the DSC 7 Differential Scanning Calorimeter and the TGA 7 Thermogravimetric Analyzer, the TMA permits the determination of dimensional and viscoelastic changes in nearly all types of sample materials. TMA, TGA, and DSC experiments can be performed simultaneously and completely independent of each other through a single PE 7500 Professional Computer.

The company continues to expand its TADS Series Thermal Analysis System with the *DSC-4 Robotic System.* Designed to be compatible with DSC-4 Differential Scanning Calorimeters, the DSC-4 RS can enhance the productivity of the thermal analysis laboratory. The robotic system makes it possible to run 48 DSC samples without operator intervention.

*Computer-aided chemistry software packages*

Several new software packages for computer-aided chemistry were demonstrated by Perkin-Elmer at Pittsburgh '85.

*LIMS 1.60*, a new information management system that allows data to be transferred between different Perkin-Elmer instruments and applications programs running on Series 7000 Professional Computers. Data and files can easily be transferred for storage, further data manipulation or archival purposes on the LIMS 2000.

*GS 2000 Graphics System* for LIMS 2000 that offers analysts five routines for curve manipulation including normalization, arithmetic, deconvolution, differentiation, and statistics. The package is completely menu-driven and special function keys are used extensively to minimize keystrokes. All results are displayed on the screen or can be sent to a printer for hard copy of the data and graphs. Each routine provides a special function for laboratory personnel to ease their work-load and increase their productivity.

*GRAPH 7000*, a general-purpose graphics program, which combines curve fitting and statistics routines for the PE 7300 and PE 7500 Professional Computers. The program is completely menu-driven which makes it easy to operate and also makes extensive use of the special function keys for simplified operation. One of the primary uses of GRAPH is the construction of calibration curves.

*DMS 7000*, a data-base program for the PE 7300 and PE 7500 Professional Computers, which provides the chemist with general data management capabilities.

Circle No. 78 on Reader Enquiry Card

### Ionics, Incorporated

*(Jana Walker, 65 Grove Street, Watertown, Massachusetts 02172; tel.: 617 926 2500)*

A remote communication system for the Digichem 3000 programmable chemical analyser was announced by Ionics. The new system is designed to permit the DigiChem 3000 user to access the DigiChem 3000 from a remote site, interrupt the operating analyser (via a password), modify or create new operating parameters (programs), restart the analyser, and monitor it for verification of correct operation. The Remote Communication System consists of hardware expansions to the DigiChem – a CPU board with expanded memory, an RS-232C board for direct communication and an optional modem for distant communications over 'phone lines, the DigiChem Remote Editor software package, communication software, and the Portable Interface Computer.

Creating or changing DigiChem operating programs is facilitated through features provided in the DigiChem Remote Editor Software package. This package features full DigiChem program editing, on-line help menus, input error detection, plain text prompts, PROM and RAM copy routines, and password protection.

The interface computer is a battery-operated portable unit which is both drip-proof and dust-tight to stand up to an industrial environment. The basic Remote Communication System will be available in June 1985.

Circle No. 79 on Reader Enquiry Card

### Chemical Sensors Club

A steering committee has been set up to consider the formation of a Chemical Sensors Club. The idea was a joint proposal by the UK's Laboratory of the Government Chemist and Warren Spring Laboratory. At a meeting attended by over 80 delegates representing a wide cross-section of industrial companies, research establishments and universities, the proposal to form a club to promote the development and application of chemical sensors was greeted enthusiastically.

*Organizations interested in participating, or requiring information on this rapidly developing area of research, should contact: Mr D. G. Porter, Laboratory of the Government Chemist, Cornwall House, Waterloo Road, London SE1 8XY.*

Circle No. 80 on Reader Enquiry Card

---

### Laboratory reactor

A new automatic laboratory reactor has now been launched in the UK by the Scientific Instruments Department of Contraves Industrial Products of Ruislip. Contalab is a computer-controlled laboratory reactor designed for day-to-day use in chemical research and development laboratories. Complete reactions can be performed and monitored automatically and a simple question-and-answer dialogue guides the operator through all process data entry procedures. The computer controls the experiment, compiles all the data, and produces a record of all results. For total safety the system automatically assigns alarm limits enabling Contalab to be used 24 h a day, completely unsupervized.

Contalab is especially suited to experiments which must be repeated many times or performed consecutively with minor changes (serial tests and optimization for example). This releases the operator from tedious monitoring and time-consuming routine work.

The system has the following main components: reactor assembly with balance (for use in a laboratory fume cupboard); control unit with dual disk drive; peripheral equipment for data input and output; and software package (for controlling system operation and processing sequences).

The *reactor assembly* is equipped as standard with a 1 l reaction vessel, a reflux condenser and a stirrer. All technical accessories are included and the following measurements can be made: temperature inside reaction vessel; jacket temperature; internal pressure in reaction vessel; pH value and weight on balance.

The *control unit* contains the measurement, control and computer electronics, as well as dual disk drive for mass data storage.

The *peripheral equipment* is used to input and output information and data and the *software package* provides the basis for interactive communication with Contalab.

The operator can access a wide range of basic operations which can be combined in many different ways. The manufacturer claims that even complex chemical processes can be performed smoothly and with high precision. Contalab will control internal temperature, add liquid, maintain pH, and handle all these functions simultaneously. Protective (inert) gas purging may be used when processing sensitive materials. Monitoring limits can be pre-set to required values. The alarm threshold may be adjusted to danger level or to any specific requirement for a given stage in the process.

*Full information on Contalab is available from the Scientific Instruments Department, Contraves Industrial Products Ltd, Times House, Station Approach, Ruislip, Middlesex HA4 8LH, UK. Tel.: 08956 30196.*

**Circle No. 81 on Reader Enquiry Card**

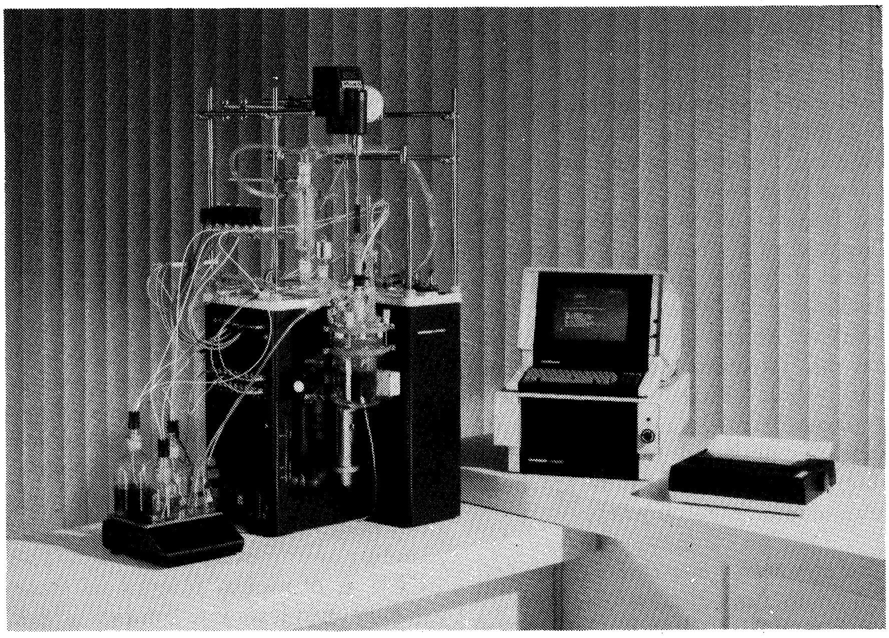

*Contraves's Contalab. Options for the system include various reactor vessels; differently shaped stirrers; phase separation and addition device for pourable solids. In support of Contalab, Contraves provide user training, introductory workshops, installation and start-up and full software support.*

### RGA data system

Vacumetrics, Inc. has announced an inexpensive data system designed specifically for computerless RGAs. This system, called Prospect, provides mass scan control from 1 s to 100 h, selective ion monitoring of up to nine peaks, partial pressure and trend analysis.

*For more information contct Kenneth Lykins at Vacumetrics, Inc., 2261 Palma Drive, Ventura, California 93003–5789, USA. Tel.: 805 644 7461.*

**Circle No. 82 on Reader Enquiry Card**

### Cyclone users' club

To promote wider appreciation of separation processes involved in cyclonic devices, Concentration Heat & Momentum (CHAM) Ltd, London, has announced the formation of a Cyclonic Separation Club in conjunction with Warren Spring Laboratory. Membership is open to individuals or to companies in user industries, as well as to cyclone designers and manufacturers, and academic bodies with interests in the field of separation. Advantages of joining the new club include cost sharing of collaborative projects, and preferential rates in the licensing and use of CHAM's software.

*Further details from Dr K. A. Pericleous, CHAM Ltd, Bakery House, 40 High Street, Wimbledon, London SW19 5AU. Tel.: 01 947 7651.*

**Circle No. 83 on Reader Enquiry Card**

### Telsec

Sieger Ltd, one of the World's largest manufacturers of gas detection equipment, has acquired Telsec Process Analysers Limited (TPA) of Peterborough from its previous owners, which include Prutec Ltd. TPA was formed five years ago by Managing Director Peter Mitchell and specializes in the manufacture of on-line process analytical instruments for the oil, chemical, food and power generating industries.

Sieger will be able to support TPA by its considerable expertise in the development of highly technical products and its outstanding success in penetrating export markets. TPA will continue to operate from its Peterborough base with its own identity and existing management.

*For further information contact Shaun Everett at Telsec Process Analysers Ltd, 34 Tresham Road, Orton Southgate, Peterborough PE2 0SE, UK. Tel.: 0733 235500.*

**Circle No. 84 on Reader Enquiry Card**

### Tablet dissolution

Tablet dissolution is an analysis widely used in the pharmaceutical industry to determine the rate at which a tablet dissolves and releases its therapeutic ingredients. This information is important for quality control of pharmaceuticals, as well as in research work on new drugs. Perkin-Elmer have introduced a completely automated system for tablet dissolution studies. L3DA consists of a Perkin-Elmer Lambda 3 UV/visible spectrophotometer, a

Model 3600 Data Station, a six cell changer and 'Dissolution' software. The double beam optics of the Lambda 3 ensure freedom from drift to achieve recording accuracy. The software included in the system is extremely powerful, yet very easy to use. It controls the entire system, logs data and prints results in a choice of seven different formats. Methods (including standard and base-line data) are stored on floppy disk, and can be retrieved at a later date. The whole L3DA system – spectrophotometer, data system and software – is designed to produce reliable and accurate results for both routine and research work.

*More information from Perkin-Elmer Ltd, Post Office Lane, Beaconsfield, Buckinghamshire HP9 1QA, UK. Tel.: 04946 6161.*

**Circle No. 85 on Reader Enquiry Card**

### Jobin-Yvon

The French instrument company, Jobin-Yvon, has been facing an uncertain future for some time, due to the failure of the steel-making firm, Creusot-Loire, which owned Instruments SA, Jobin-Yvon's parent company. Jobin-Yvon are world leaders in optical research, and application to instruments. They marketed the first polarimeter, the first Fabry-Perot interferometer, the first concave holographic gratings, and have many patents in optics. Their instruments include spectrometers (ICP, spark, Raman, etc.), circular dichrographs, gratings, monochromators, and others including preparative liquid chromatography.

The news has just been released that a management buyout of the company has been successful. This is backed by Jobin-Yvon's banks, private investors, and venture capitalists. Both I.S.A., Inc. of the US, and I.S.A. GmbH of FR Germany remain as wholly owned subsidiaries. The new investment and loan capital will increase the company's capital by 65 million francs (£6 million), and they plan major research and development efforts in instrumentation.

EDT Research of London, the UK agents, will continue to supply and service the Jobin-Yvon range.

*Details from EDT Research, 14 Trading Estate Road, London NW10 7LU. Tel.: 01 961 1477.*

**Circle No. 86 on Reader Enquiry Card**

### Liquids dispensing

A liquids process timer, which automates the turning off and on of liquids, has been announced by Datronix Controls. The new timer, called the PT41, is claimed to offer the most comprehensive performance of any such process timer currently on the market. It is suited for a range of tasks in many industries – from automatically carrying out boiler blow-down operations, to cooling tower-basin 'bleeding'. The PT41 is of special interest to those dosing chemicals into solution which are difficult to measure by PH or conductivity. It will control automatically the addition of chemicals, fresh water or any other liquid into any type of system.

The PT41 will activate puimps on and off for any period and with any frequency required. There are two ranges of 'on' times: from 0.1 to 1.0 min and from 1.0 to 10.0 min. 'Off' times are adjustable from 0.5 to 5.5 h. When first switched on the timer goes immediately to the 'on' mode, showing an amber indicator light, and thereafter cycles between the preset on and off periods continuously. An accuracy of 5% of the dispensed medium is achieved with repeatability accuracy time of 2%. A fused on-off power switch is provided and the unit housing is a moulded blue case fully gasketed and fitted with waterproof cable glands.

*Information from the Sales Office, Datronix Controls Limited, Datronix House, Lower Kings Road, Berkhamsted, Hertfordshire HP4 2AE, UK. Tel.: 04427 73377.*

**Circle No. 87 on Reader Enquiry Card**

### Silver recovery

MCP Holding's group of companies who have a long history of refining high purity specialized metals has entered the silver recovery market following their acquisition of the Metelec Co. from Wilson Process Systems Ltd. The new company will be called MCP-Metelec Ltd and will initially be providing its services to radiography departments, photo-finishing, lithographic and printing industries. The company will be offering customers a range of options which include either the supply of recovery equipment, the refining of silver-bearing material from existing systems or a total package which includes supply of equipment and collection and treatment of recovered silver on behalf of the customer.

*More information from MCP-Metelec Ltd, Middlesex, HA0 4PE, UK*

**Circle No. 88 on Reader Enquiry Card**

### Canberra is distributing Mentor

Mentor process management and control software is for traditional batch and continuous processes in the food, pharmaceutical, chemical, plastics, pulp and paper industries. Small production companies and pilot plant facilities can be automated using a single IMACS processor and Mentor software. The Mentor software package, as implemented on the IMACS System 90, offers a flexibile process interface. The system can interact directly with the process via a wide variety of analogue and digital signal conditioning hardware including thermocouple, RTD, strain gauge, current loop, and high or low voltage discrete signals. Mentor also has the ability to interface to a variety of intelligent control devices via RS-232C and RS-422C serial communications links (Canberra believe that this is unique to Mentor).

Several programmable controllers and loop controllers, as well as Canberra's System 50 remote control unit, are supported as standard interfaces to the system. The software structures intrinsic in Mentor allow for fully consistent operation and operator interface. The power of the serial interfaces allows a distributed system architecture to be constructed to control larger-scale applications.

Process control schemes are implemented assigning algorithms to process input/output points; 30 standard control algorithms are available and the user can also write and store nearly 100 other algorithms.

The Mentor software package simultaneously maintains three historical data-bases; continuous history, unit history, and alarm/function change history.

The continuous history data-base stories historical data for up to 16 data classes. The unit history database allows for historical data to be grouped by process units. Spot values are collected periodically, sometimes as often as every 10 s. The collection period is operator accessible and can be adjusted to suit the needs of particular operating conditions. The operator can also initiate the 'demand' collection, which will store spot values, allowing the operator to take a 'snapshot' of operating conditions. Operator comments and sequence initiated messages are also stored in unit history, allowing for an accurate analysis of operating conditions. The Alarm/Function Change (AFC) history maintains a circular file of all alarms, events and operator interactions in chronological order. The AFC history can be sorted for display or print-out. Alarm priorities may be set to initiate 'shut down sequences', for example discharging a 'spoiled' batch.

Canberra has developed an advanced operator interface system for the Mentor package which utilizes the IMACS high-resolution colour graphic display with a touch-sensitive screen overlay.

The operator can page through displays of increasing levels of detail, zooming in on the process parameter of particular interest. In addition to menu, faceplate profile, single loop detail and trend displays, the system offers batch summary and batch unit displays allowing the operator to closely monitor sequence execution. Operator control of sequence operation, setpoint and alarm limit access, and parameter modification, all occur through touchscreen interaction.

*Details from Canberra Instruments Ltd, Block D, The Dorcan Complex, Faraday Road, Dorcan, Swindon, Wiltshire SN3 5HX, UK. Tel.: 0793 35384.*

**Circle No. 89 on Reader Enquiry Card**

### Monitoring suspended solids

Dissimilar materials and suspended solids in liquid streams with concentrations down to parts per billion can be monitored continuously online with the MPS-3000 system from Micro Pure Systems. This is available in the UK from Auriema and features non-invasive ultrasonic measurement with sensors that mount directly and easily onto process pipelines. Measurement from ppb to percentage levels is independent of flow rate or process stream colour and avoids problems such as lamp ageing, alignment and filming, which are commonly met with optical systems.

The MPS-3000 monitors up to six sensing points, typically for checking flow downstream of a bank of filters or suspended solids contamination levels at a series of points in a process. If a reading reaches a preset level, an audible alarm is sounded and the computer controller prints-out locations, time and upset level at the sensing point. An external alarm operates via an IEEE-488 output from the monitor; and the MPS-3000 provides a menu of report formats including 4 and 24 h graphs and tables as well as one week's data storage on magnetic tape.

Also available shortly will be a single-channel system with a 4-20 mA output.

*More information from Auriema Ltd, 442 Bath Road, Slough SL1 6BB, UK. Tel.: 06286 4353.*

**Circle No. 90 on Reader Enquiry Card**

### ZyMOS and Mentor and circuit design

Following an agreement between ZyMOS Corporation, California and Mentor Graphics Corporation, Oregon, the ZyP standard-cell libraries for VLSI circuit design are now available for use with Mentor's IDEA 1000 workstations. In the UK, the ZyP system is marketed by Chiptech Ltd. The ZyP libraries offer pre-defined standard logic functions that are used in the design of integrated circuits based on 5- or 3-micron silicon gate CMOS technology. The cells vary in complexity from simple logic gates to RAM, ROM, PLA core microprocessor and analogue functions. The libraries now include capabilities, designed specifically around the Mentor Graphic hardware and software, which enable designers to use Mentor's schematic capture, logic simulation and verification software. A 'net list extractor' contains all the information on how cells are interconnected, and extractor software automatically generates a ZyP-compatible net list from the Mentor Graphics net list. Using this ZyP net list, Chiptech can produce the chip, having eliminated the risk that a custom circuit may not function. Further advantages to using the ZyP Libraries in chip design include reduced design and development costs and a shorter development cycle.

*Enquiries to Elgan Howell, Chiptech Ltd, Tewin Court, Welwyn Garden City, Hertfordshire, UK. Tel.: 07073 32140.*

**Circle No. 91 on Reader Enquiry Card**

### New Hewlett-Packards

*Automated Headspace Sampler for preparing solid or viscous liquid samples for syringe injection to packed or capillary gas chromatography or GC/MS systems*

Laboratories in the petrochemical, plastic, food, cosmetics and many other industries frequently need to analyse organic volatiles that are bound within a solid matrix or a liquid with high solids content. Printing inks, plastic food containers, anti-perspirant sticks and pollutants in soils and sludges are examples. Such samples cannot be injected onto the GC colum without preparation. The HP 19385A greatly reduces this sample preparation time. The untreated sample is placed in a 10 or 20 ml glass vial, which is sealed with a septum and crimped cap and placed in the instrument's 24-vial carousel. Heating the vials in a silicone oil bath drives the vapour from the sample. The vapour is collected by a syringe through the septum, or a heated transfer line is used to carry it directly onto the column. The volume of vapour injected is accurately controlled and highly reproducible with typical relative standard deviations (RSD) of better than 1%. Instrument performance for the analysis of aqueous chlorinated hydrocarbons at the 1 ppb is less than 5% RSD.

Four sets of user-defined operating conditions may be stored in the sampler, allowing analyses to be automated. The sampler operates over the temperature range from 15 to 150 °C. Since the vapour in head-space analysis is much 'cleaner' than a liquid sample, routine injection port cleaning is required less often and GC columns last longer.

To achieve optimum qualitative and quantitative results, Hewlett-Packard recommends a system comprising the HP19395A Headspace Sampler on an HP5880A Gas Chromatograph equipped with an HP5970A Mass Selective Detector.

*HP7673A automatic injector for laboratories requiring high chromatographic precision but which do not need large-volume sample throughput*

The injector handles an automatic sequence of up to three liquid samples, with injection parameters regulated by the HP7673A's built-in controller, or through the HP3392A integrator or the HP5880A gas chromatograph. It may also be configured into a fully automated laboratory system, linking into the HP instrument network (INET) integrator with the HP5890A gas chromatography may then be stored. As integrator files may be linked, different sample types can be analysed using variable GC and injector methods. This capability may be further expanded by networking the integrator into the HP3350 laboratory automation system.

The HP7673A brings a new standard of accuracy and reproducibility to the injection technique, because the sample is introduced into the chromatograph at high speed, minimal heat is transferred from the injection port to the stainless-steel syringe needle, making it unlikely that sample discrimination will occur from syringe effects. The overall precision of 0·5% ensures that sample composition is not altered by inconsistencies in the injection technique.

Operation convenience and simplicity were major considerations in the design of the HP7673A. Its compact size allows it to be installed or removed from the gas chromatograph in seconds, by a simple twist of the wrist. It aligns itself automatically, and alignment adjustments are unnecessary as the syringe moves only in a vertical direction with the samples rotating beneath it. Fault indicator lamps provide error codes for easy troubleshooting.

The on-column capability of the HP7673A, which was designed for use with 530 micron columns, can be adapted easily to standard fused silica capillary columns by using a column connector. To reconfigure the HP7673A for automatic on-column injection, a single command is entered – no hardware changes are needed.

Hewlett-Packard expects the HP7673A to find many applications in quality control, research and methods development in such industries as pharmaceuticals, food and flavours, and in environmental research.

*Reader's enquiries to Enquiry Section, Hewlett-Packard Ltd, Eskdale Road, Winnersh, Wokingham, Berkshire, UK.*

**Circle No. 92 on Reader Enquiry Card**

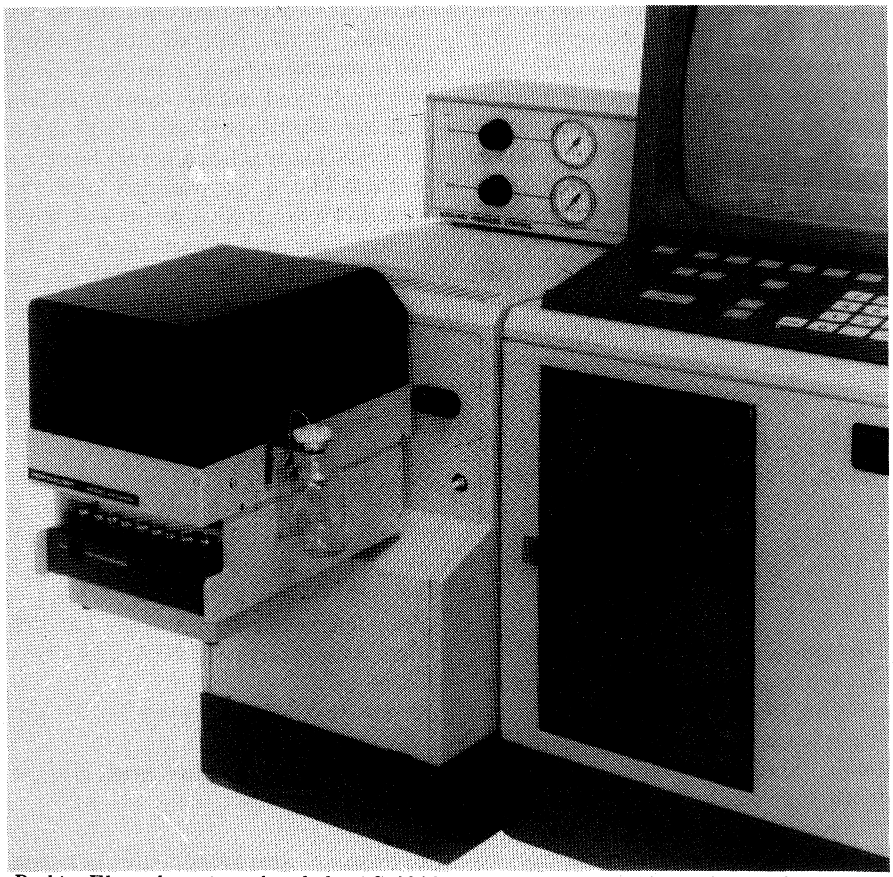

*Perkin-Elmer have introduced the AS-8300, a new programmable, multisampling system for their Model 8300 gas chromatograph. The AS-8300/Model 8300 combination is offered as a versatile and cost-effective liquid autosampling system. It will be particularly useful for process or quality-control applications in manufacturing environments involving routine analyses of a very wide range of liquid samples—from pharmaceuticals, petrochemicals and pesticides to foods, aromas and biological fluids. The AS-8300 consists of a pneumatically operated injection system and electronically controlled, removable sample tray that holds up to 100 vials. It is operated from the keyboard and VDU of the Model 8300. Up to 10 sampling sequences can be generated for the AS-8300 which are completely interactive with any of 10 GC methods that can be stored in the memory of the 8300; a sampling sequence can consist of up to 10 different groups of samples. Sampling and injection parameters, such as the number of injections per vial, flush cycles and injection times, are selectable for each group of samples. A sequence can be 'paused' at any time to allow a single sample to be interposed without disrupting the original sequence, and deviations are reported at the end of every sequence. Perkin-Elmer Ltd can be contacted at Post Office Lane, Beaconsfield, Buckinghamshire HP9 1QA, UK.*

**Circle No. 93 on Reader Enquiry Card**

